# Linking Biomedical Data Warehouse Records With the National Mortality Database in France: Large-scale Matching Algorithm

**DOI:** 10.2196/36711

**Published:** 2022-11-01

**Authors:** Vianney Guardiolle, Adrien Bazoge, Emmanuel Morin, Béatrice Daille, Delphine Toublant, Guillaume Bouzillé, Youenn Merel, Morgane Pierre-Jean, Alexandre Filiot, Marc Cuggia, Matthieu Wargny, Antoine Lamer, Pierre-Antoine Gourraud

**Affiliations:** 1 CHU de Nantes, INSERM CIC 1413 Pôle Hospitalo-Universitaire 11: Santé Publique, Clinique des données, 44000 Nantes France; 2 LS2N UMR CNRS 6004 Université de Nantes - 2, rue de la Houssinière - BP 92208 - 44322 Nantes Cedex 03 - France Nantes France; 3 Univ Rennes, CHU Rennes, INSERM, LTSI-UMR 1099,35000 Rennes France; 4 CHU Lille, INCLUDE: Integration Center of the Lille University Hospital for Data Exploration, 59000 Lille France; 5 Univ. Lille, CHU Lille, ULR 2694 METRICS: Évaluation des Technologies de santé et des Pratiques médicales, F-59000 Lille France; 6 Université de Nantes, CHU de Nantes, INSERM Centre de Recherche en Transplantation et Immunologie, UMR 1064, ATIP-Avenir Nantes France

**Keywords:** data warehousing, clinical data warehouse, medical informatics applications, medical record linkage, French National Mortality Database, data reuse, open data, R, clinical informatics

## Abstract

**Background:**

Often missing from or uncertain in a biomedical data warehouse (BDW), vital status after discharge is central to the value of a BDW in medical research. The French National Mortality Database (FNMD) offers open-source nominative records of every death. Matching large-scale BDWs records with the FNMD combines multiple challenges: absence of unique common identifiers between the 2 databases, names changing over life, clerical errors, and the exponential growth of the number of comparisons to compute.

**Objective:**

We aimed to develop a new algorithm for matching BDW records to the FNMD and evaluated its performance.

**Methods:**

We developed a deterministic algorithm based on advanced data cleaning and knowledge of the naming system and the Damerau-Levenshtein distance (DLD). The algorithm’s performance was independently assessed using BDW data of 3 university hospitals: Lille, Nantes, and Rennes. Specificity was evaluated with living patients on January 1, 2016 (ie, patients with at least 1 hospital encounter before and after this date). Sensitivity was evaluated with patients recorded as deceased between January 1, 2001, and December 31, 2020. The DLD-based algorithm was compared to a direct matching algorithm with minimal data cleaning as a reference.

**Results:**

All centers combined, sensitivity was 11% higher for the DLD-based algorithm (93.3%, 95% CI 92.8-93.9) than for the direct algorithm (82.7%, 95% CI 81.8-83.6; *P*<.001). Sensitivity was superior for men at 2 centers (Nantes: 87%, 95% CI 85.1-89 vs 83.6%, 95% CI 81.4-85.8; *P*=.006; Rennes: 98.6%, 95% CI 98.1-99.2 vs 96%, 95% CI 94.9-97.1; *P*<.001) and for patients born in France at all centers (Nantes: 85.8%, 95% CI 84.3-87.3 vs 74.9%, 95% CI 72.8-77.0; *P*<.001). The DLD-based algorithm revealed significant differences in sensitivity among centers (Nantes, 85.3% vs Lille and Rennes, 97.3%, *P*<.001). Specificity was >98% in all subgroups. Our algorithm matched tens of millions of death records from BDWs, with parallel computing capabilities and low RAM requirements. We used the Inseehop open-source R script for this measurement.

**Conclusions:**

Overall, sensitivity/recall was 11% higher using the DLD-based algorithm than that using the direct algorithm. This shows the importance of advanced data cleaning and knowledge of a naming system through DLD use. Statistically significant differences in sensitivity between groups could be found and must be considered when performing an analysis to avoid differential biases. Our algorithm, originally conceived for linking a BDW with the FNMD, can be used to match any large-scale databases. While matching operations using names are considered sensitive computational operations, the Inseehop package released here is easy to run on premises, thereby facilitating compliance with cybersecurity local framework. The use of an advanced deterministic matching algorithm such as the DLD-based algorithm is an insightful example of combining open-source external data to improve the usage value of BDWs.

## Introduction

Vital status is important information for medical research. While real-world evidence from the analysis of biomedical data warehouse (BDW) records has gained popularity in recent years [1], the longitudinal value of the information is weakened by the uncertainty of patients’ vital statuses. This information is often limited to inpatients who died while hospitalized.

France has a long tradition of administrative centralization inherited from the Napoleonian era. When a resident dies on French territory, French city halls are required to send a death report to the Institut National de la Statistique et des Études Économiques (INSEE), translated to the French National Institute of Statistics and Economic Studies [2]. This report is used to complete the French National Mortality Database (FNMD). This database, which contains tens of millions of records, is updated monthly and has been open access since 2019 [3].

Record linkage (also referred to as data matching or entity resolution) is the process of quickly and accurately identifying records corresponding to the same individual entity from one or more data sources [4]. A recent literature review by Bounebache et al [5] presents record linkage and its multiple challenges. Two different approaches exist: (1) deterministic record linkage, which uses expert knowledge and possible statistical learning [5]; and (2) probabilistic linkage, which relies on a statistical model to evaluate the contribution of each variable in the record linkage strategy [6,7]. Matching large-scale BDW records with the FNMD presents multiple challenges. The first is the absence of a unique common identifier, such as a social security number. Second, surnames are shared within families and may change over one’s lifetime based on varying cultural practices regarding marriage. Additionally, first and middle names can be confounded or compound. Third, clerical errors can occur when identities are administratively recorded in both databases [4]. Furthermore, in practice, the exponential number of comparisons often prohibits direct matching of millions of database records to the FNMD, which contains tens of millions of records.

Consequently, little has been published on the computational performance of record linkage, its accuracy, and its determinants. Moore et al [8] state that record-linkage performance must be evaluated to validate statistical analyses. For example, they showed that a specificity of <95% prevents estimating a significant risk ratio of 2. Previous studies [9,10] have used record linkage with the FNMD. Most of these studies used suboptimal references to evaluate algorithm performance, small databases (ie, <20,000 patients), were monocentric, or did not share their source code. Bannay et al [11] linked a BDW with Système National des Données de Santé (SNDS), translated to the French National Health Database. As the extraction from the SNDS was anonymized in accordance with national legislation, they implemented a semideterministic record linkage procedure based on the variables of the hospital discharge report (ie, sex, year of birth, month of birth, admission and discharge dates, diagnoses, etc).

To routinely update vital status in BDW records from the FNMD on a large scale, we developed a deterministic matching algorithm based on Damerau-Levenshtein distance (DLD) and compared its performance with that of a direct-matching algorithm as a reference for 3 regional hospital BDWs.

## Methods

### Data and Databases

FNDM files were downloaded from the national open data website [12] and included the following fields: birth surname, first name, middle names, birth date, sex, city and country of birth, death date, and zip code of the place of death. We found 11,490,867 records for the period between 2001 and 2020.

Three university hospitals in France were involved in this study: Lille, Nantes, and Rennes. Each hospital’s BDW contains administrative, clinical, biological, and drug data.

In the Lille BDW, vital status information was available on June 1, 2021, for 1,609,515 patients who had at least 1 hospital stay between January 1, 2008, and June 1, 2021. The data showed that 1,570,320 (98%) patients were living, and 39,195 (2%) were deceased.

For the Nantes BDW, vital status information was available on January 14, 2021, for 2,035,805 patients who had at least 1 hospital encounter during the previous 20 years. The data showed that 1,974,786 (97%) patients were living and 61,019 (3%) were deceased.

For the Rennes BDW, vital status information was available on January 4, 2021, for 1,262,072 patients. The data showed that 1,221,817 (97%) patients were living, 37,986 (3%) were deceased, and 346 had no recorded vital status.

Hereafter, samples extracted from the BDWs are referred to as “local databases.”

### Record-Linkage Algorithms

To assess the performance gain induced by advanced data cleaning and DLD use, we used a simple direct-matching algorithm as a reference. Characteristics of the data cleaning and the algorithms used are presented in Multimedia Appendix 1.

### Direct-Matching Algorithm as a Reference

The direct-matching algorithm removed accents from patients’ first name and surname because the FNMD does not use accents. All letters were transformed into lowercase. Two records were linked between the local database and the FNMD if both records had the exact same surname, first name, birth date, and sex. The surname chosen was the birth surname if present or the current surname if not.

### DLD-Based Algorithm as a Deterministic Solution

The DLD between 2 strings is the sum of necessary operations to transform string 1 into string 2, among insertion of a character, deletion of 1 character, substitution of 1 character by another, or transposition of 2 adjacent characters [13]. Examples are available in Multimedia Appendix 2.

Distances were calculated between the local database and FNMD for first name, surname, birth date, sex, and city of birth. For sex, a distance of 0 indicates that the sex is the same in both records, and a distance of 1 indicates a mismatch between the 2 records.

### A 4-Step Algorithm

The algorithm can be divided into four consecutive steps: cleaning the data, creating new variables, validating pairs with blocking techniques, and choosing the more pertinent pairs.

#### Data Cleaning

In the FNMD, birth dates may be expressed with a missing day and month (eg, 1956-00-00). In these cases, the algorithm automatically attributed January 1 to the date to obtain a valid date format. If the birth date was invalid, the month and day were inverted and tested before choosing January 1. For example, the date 1960-31-03 is invalid (ie, there are not 31 months in a year); however, the date 1960-03-31 is valid, so 1960-03-31 was chosen. Another example is the date 1959-32-33: the dates 1959-32-33 and 1959-33-32 are also invalid; thus, the date 1959-01-01 was chosen.

Characters other than letters (eg, numbers and special characters) were removed from the local database and FNMD. All letters were changed to lowercase, and accents were removed.

In both the local database and FNMD, mentions of the district were suppressed, and only the city of birth was used. For example, “Paris, 13ème arrondissement” was changed “paris.”

#### New Variable Creation

In the local database, a transformed city of birth variable was created wherein abbreviations were transformed into full text. For example, “St-Martin-sr-Ocre” was transformed into “saintmartinsurocre.”

In the FNMD, the variable fnmd_firstname_0 was created from the first element of the first name (eg, “pierre” from “Pierre-Olivier”), and the variable fnmd_firstname_12 was created from concatenation of the first name and middle name (eg, “marieclaire” from first name “Marie” and middle name “Claire”). Other examples are available in Table 1.

**Table 1:** Examples of first name–related data created for first name Damerau-Levenshtein distance (DLD) computation.

Original data from the FNMD^a^		Data created for first name DLD^b^ computation		
First name	First middle ame	fnmd_firstname_0	fnmd_firstname _1	fnmd_firstname_12
Jean	N/A^c^	jean	Jean	jean
Marie	Claire	marie	marie	marieclaire
Pierre-Olivier	Christian	pierre	pierreolivier	pierreolivierchristian
Elon-Louis	N/A	elon	elonlouis	elonlouis

^a^French National Mortality Database.

^b^Damerau-Levenshtein distance.

^c^Not applicable.

#### Pair Validation

Records from the local database and the FNMD matched if all the following conditions were valid: (1) the DLD of the first name was ≤ the maximal first name DLD, (2) the DLD of the surname was ≤ the maximal surname DLD, (3) the DLD of the birth date was ≤ the maximal birth date DLD, (4) the DLD of the sex was ≤ the maximal sex DLD, and (4) the total sum of the 4 previous DLDs (ie, the total DLD) was ≤ the maximal of the total DLD.

The DLD chosen for the surname was the shorter DLD among (1) the birth surname in the local database and the surname in the FNMD and (2) the current surname in the local database and the surname in the FNMD.

The DLD chosen for the first name was the shorter DLD among (1) the first name in the local database and fnmd_firstname_0, (2) the first name in the local database and fnmd_firstname _1, and (3) the first name in the local database and fnmd_firstname_12.

The DLD chosen for the birth city name (option for the more pertinent pairs selection) was the shorter DLD among (1) the original city of birth in the local database and city of birth in the FNMD and (2) the transformed city of birth in local database and city of birth in the FNMD.

Matching 2 databases (A and B) without a common identifier implies evaluating the match or nonmatch status of every element of AxB, called a pair [5]. The number of pairs to compare is given by the number of records in A multiplied by the number of records in B. This formula is particularly concerning when matching BDWs with the FNMDs, both of which potentially contain tens of millions of records, potentially leading to quadrillions of pairs to compare. Blocking techniques reduce the number of pairs to compare [5] and thus the execution time and RAM requirements. We successively applied a simple blocking technic 2 times, which consist of only comparing the pairs that contained the same value for 1 defined variable: first, the birth date and second, the concatenation of the first 4 characters of the first name and the first 4 characters of the surname (the birth surname when present and the current surname elsewhere). This allowed us to process the pairwise comparison, even if the birth date or the first 4 characters of first name/surname contained mismatches (but not if both birth date and the first 4 characters of the first name/surname contained mismatches).

Table 2 presents examples of pairs going through comparison process or not, given the 2 successive blocking processes used in the DLD-based matching algorithm.

**Table 2:** Examples of the blocking process for the Damerau-Levenshtein distance (DLD)–based matching algorithm.

FNMD^a^ birth date	FNMD first name/surname concatenation^b^	Local database birth date	Local database first name/surname concatenation^b^	Comparison during birth date blocking	Comparison during first name/family name concatenation blocking
1935-06-29	louidefu	1935-06-29	louidefu	Yes	Yes
1935-06-29	louidefu	1931-10-08	maricall	No	No
1935-06-29	louidefu	1940-26-11	jeanpoku	No	No
1935-06-29	louidefu	1956-23-12	chardegu	No	No
1956-12-18	maricall	1935-06-29	louidefu	No	No
1956-12-18	maricall	1931-10-08	maricall	No	Yes
1956-12-18	maricall	1940-26-11	jeanpoku	No	No
1956-12-18	maricall	1956-23-12	chardegu	No	No
1940-11-26	jeanpoqu	1935-06-29	louidefu	No	No
1940-11-26	jeanpoqu	1931-10-08	maricall	No	No
1940-11-26	jeanpoqu	1940-11-26	jeanpoku	Yes	No
1940-11-26	jeanpoqu	1956-23-12	chardegu	No	No
1940-11-26	maricuri	1935-06-29	louidefu	No	No
1940-11-26	maricuri	1931-10-08	maricall	No	No
1940-11-26	maricuri	1956-23-12	chardegu	No	No
1940-11-26	maricuri	1956-23-12	chardegu	No	No

^a^FNMD: French National Mortality Database.

^b^Concatenation of the 4 first characters of the first name and the 4 first characters of the surname (birth surname if present, current surname elsewhere).

#### Choice of More Pertinent Pairs

One patient from a local database could be matched with none, 1, or multiple records from the FNMD. An algorithm is proposed in Multimedia Appendix 3 to select the most pertinent pairs for the last cases.

### Data Sampling for Statistical Learning, Performance Evaluation, and Large-scale Testing

For all individuals, the following variables were extracted: birth surname, current surname, first name, birth date, sex, city and country of birth, vital status, and, if present, death date.

To learn the optimal parameters of the DLD-based algorithm, we randomly selected 3600 patients from the Nantes BDW, with 450 per stratum: (1) men born in France (MBIF) who died between 2001 and 2020 (deceased MBIF). These were the MBIF whose deaths were registered in the BDW between 2001 and 2020; (2) MBIF alive on 1 January 2016 (living MBIF). These were the MBIF with at least 1 hospital encounter between January 1, 2001, and December 31, 2015, and another hospital encounter between January 2, 2016, and December 31, 2020; (3) women born in France (WBIF) who died during between 2001 and 2020 (deceased WBIF); (4) WBIF who were alive on January 1, 2016 (living WBIF); (5) men born outside France (MBOF) who died between 2001 and 2020 (deceased MBOF); (6) MBOF who were alive on January 1, 2016 (living MBOF); women born outside France (WBOF) who died between 2001 and 2020 (deceased WBOF); and (7) WBOF who were alive on January 1, 2016 (living WBOF).

For the DLD-based algorithm, a maximal DLD of 2 was learned for the first name, 1 for the surname, 1 for the birth date, 1 for sex, and 2 for the total DLD.

To evaluate the specificity and sensitivity of both the DLD-based and direct algorithms, samples of 8000 patients were randomly extracted from each of the 3 BDWs. The sample from Nantes did not contain patients used for statistical learning of the DLD-based algorithm parameters (ie, the maximal DLDs). Each sample contained 1000 deceased MBIF, 1000 living MBIF, 1000 deceased WBIF, 1000 living WBIF, 1000 deceased MBOF, 1000 living MBOF, 1000 deceased WBOF, and 1000 living WBOF.

Finally, to assess the low RAM requirements and parallel processing capabilities of the DLD-based algorithm, a sample of 2 million patients was randomly extracted from the Nantes BDW, and 11 million records (between 2001 and 2020) were randomly extracted from the FNMD.

### Specificity and Sensitivity Evaluated on Separate Data Sets

Sensitivity and specificity were evaluated for 8000 patients from every hospital for both the direct and DLD-based algorithms. Sensitivity (or recall) was evaluated for patients who died between January 1, 2001, and December 31, 2020, and were registered as the gold standard for each BDW. The algorithm classified patients as deceased if they were linked by at least one FNMD record. Specificity was evaluated for patients alive on 1 January 2016 (ie, patients with at least 1 hospital encounter between January 1, 2001, and December 31, 2015, and another hospital encounter between January 2, 2016, and December 31, 2020). The algorithm classified a patient as alive if the patient was not linked to the FNMD. The same patient could be present in both the sensitivity and specificity data sets (eg, a patient who died on May 13, 2017). This was not a problem because to evaluate specificity, we only used deaths registered in the FNMD between January 1, 2001, and January 1, 2016. To evaluate sensitivity, we used all deaths registered in the FNMD between January 1, 2001, and December 31, 2020. Specificity and sensitivity were calculated for each maximal total distance parameter of the DLD-based algorithm, from 0 to 5.

Our gold standard for the matching algorithms was for it to be reliable both for sensitivity and specificity evaluation. First, it was completely independent from the FNMD. Second, deceased status in the hospital databases was reliable because vital status at discharge is a necessary information for the stay fee payment to the hospital by public health insurance in France. Finally, alive status at a certain time was also reliable because it was searched for between 2 distinct encounters.

Global performances, global performance per hospital, performances per sex and per hospital, and performances per country of birth and per hospital were calculated using the stratified sampling proportion method. To calculate these performances, we needed the percentages of patients born outside of France for the 3 cities. French national census data for 2012 [14] yielded 4.5% (40,394/897,639) for Nantes, 4.3% (29,697/690,618) for Rennes, and 8.4% (97,988/1,166,527) for Lille. For this calculation, we considered half of the population to be composed of men and the other half of women.

### Implementation and Execution Time Evaluation

We developed an R package to run on parallel cores and automatically select by default the most efficient number of cores to use depending on the number of records to match, the number of available cores, and the available RAM. The number of cores used still fit in the parameters. We used the packages “future” and “future.apply” to enable Linux and Windows compatibility. We measured the execution time to successively match 200, 2000, 20,000, 200,000, and 2 million patients from the Nantes BDW with 11 million records from the FNDM. We tested various core numbers on 3 cores and 15 GB of RAM (1 core and 1 GB of RAM on the laptop used were left free for the operating system).

### Ethical Considerations

Each of the 3 BDWs had a previous authorization from the National Information Science and Liberties Commission. These authorizations included data quality controls that our algorithm contributes to.

## Results

### Performances of the Matching Algorithms

Table 3 compares the performances between the direct and DLD-based algorithms for all 3 hospitals combined. Sensitivity of the DLD-based algorithm was 11% higher than that of the direct algorithm (93.3%, 95% CI 92.8-93.9 vs 82.7%, 95% CI 81.8-83.6; *P*<.001). Specificity of the DLD-based algorithm was <1% lower than that of the direct algorithm (99%, 95% CI 98.7-99.2 vs 99.9%, 95% CI 99.8-100; *P*<.001). Table 4 presents overall performances by hospital for both algorithms. Sensitivity of the DLD-based algorithm for the Rennes and Lille samples was 12% higher than that for the Nantes sample (85.3%, 95% CI 83.8-86.8 vs 97.3%, 95% CI 96.7-97.9; *P*<.001). Specificity of the DLD-based algorithm was >98% in all samples (98.2% to 99.4%; *P*<.001).

Table 5 presents the performances of the DLD-based algorithm per sex and per hospital. In Lille, sensitivity was equal for both sexes (97.3%; *P*>.99). Sensitivity was higher for men than for women in Nantes (87%, 95% CI 85.1-89.0 vs 83.6%, 95% CI 81.4-85.8; *P*=.006) and in Rennes. In all hospitals, specificity for women (98.6% to 99.6%) was higher than that for men (97.9% to 99.2%), but with no statistically significant differences (*P*>.05).

Table 6 presents performances of the DLD-based algorithm per birth country and per hospital. For every hospital, sensitivity of the DLD-matching algorithm was ~10% higher (*P*<.001) for people born in France than for people born outside France (Nantes: 85.8%, 95% CI 84.3-87.3 vs 74.9%, 95% CI 72.8-77.0; *P*<.001). Specificity was >98% for every sample (range 98.2%-99.8%). In Lille, specificity was equal for people born both in and outside of France (99.4%, 95% CI 99-99.7; *P*<.99). In Nantes and Rennes, specificity for people born out of France (98.8%) was higher than that for people born in France (98.2% to 99.3%; *P*<.05).

**Table 3:** Global performances of the Damerau-Levenshtein distance (DLD)–based algorithm versus that of the direct-matching algorithm.

Matching algorithm	Sample size, N	Sensitivity, % (95% CI)	Specificity, % (95% CI)
Distance-based^a^	21860	93.3 (92.8-93.9)	99 (98.7-99.2)
Direct	21860	82.7 (81.8–83.6)	99.9 (99.8-100)
*P* value McNemar test	N/A^b^	<.001	<.001

^a^Maximal total distance: 2.

^b^N/A: not applicable.

**Table 4:** Global performances of the Damerau-Levenshtein distance (DLD)–based algorithm versus the direct-matching algorithm per university hospital.

University hospital	Total sample, n	Patients born outside of France used for weights, %	Se^a^ DLD^b^, % (95% CI)	Se direct^c^, % (95% CI)	Sp^d^ DLD, % (95% CI)	Sp direct, % (95% CI)
Nantes	Se: 3660 Sp: 4000	4.5	85.3 (83.8-86.8)	74.6 (72.8-76.4)	99.3 (99-99.7)	99.9 (99.7-100)
Rennes	Se: 2500 Sp: 4000	4.3	97.3 (96.7-97.9)	86.0 (84.6-87.4)	98.2 (97.7-98.8)	100 (99.9-100)
Lille	Se: 3700 Sp: 4000	8.4	97.3 (96.8-97.9)	87.5 (86.2-88.8)	99.4 (99-99.7)	99.9 (99.8-100)
*P* value Fisher exact test	N/A^e^	N/A	<.001	<.001	<.001	.01

^a^Se: sensitivity.

^b^DLD: Damerau-Levenshtein distance–based matching algorithm (Maximal total distance used: 2).

^c^Direct: direct-matching algorithm.

^d^Sp: specificity.

^e^N/A: not applicable.

**Table 5:** Performances of the Damerau-Levenshtein distance (DLD)–based matching algorithm by sex and university hospital.

University hospital	Total sample	Se^a^ women, % (95% CI)	Se men, % (95% CI)	*P* value Fisher exact test	Sp^b^ women, % (95% CI)	Sp men, % (95% CI)	*P* value Fisher exact test
Nantes	Se women: 1660 Se men: 2000 Sp women: 2000 Sp men: 2000	83.6 (81.4-85.8)	87 (85.1-89)	.006	99.4 (99-99.9)	99.2 (98.7-99.8)	.57
Rennes	Se women: 1300 Se men: 1200 Sp women: 2000 Sp men: 2000	96 (94.9-97.1)	98.6 (98.1-99.2)	<.001	98.6 (97.8-99.3)	97.9 (97.0-98.8)	.12
Lille	Se women: 1700 Se men: 2000 Sp women: 2000 Sp men: 2000	97.3 (96.5-98.1)	97.3 (96.6-98)	>.99	99.6 (99.2-100)	99.1 (98.6-99.6)	.08

^a^Se: sensitivity.

^b^Sp: specificity.

Finally, use of the DLD was more efficient for women and people born outside France than for men and people born in France. In Nantes, an increase from 0 to 2 for the maximal total DLD increased the sensitivity by 1.85% for MBIF, 4.4% for MBOF, 2.9% for WBIF, and 6.6% for WBOF. Performances per sex, birth country, and maximal total DLD for the DLD-based algorithm are available for Nantes hospital in Multimedia Appendix 4.

Details on the performances per strata and repartition of the DLD of the valid (sensitivity) and invalid (specificity) pairs are available for Nantes hospital in Multimedia Appendices 5-6.

**Table 6:** Performances of the Damerau-Levenshtein distance (DLD)–based matching algorithm per birth country and per university hospital.

University hospital	Sample size	Se^a,b^ BIF^c^, % (95% CI)	Se^b^ BOOF^d^, % (95% CI)	*P* value Fisher exact test	Sp^eb^ BIF, % (95% CI)	Sp^b^ BOOF, % (95% CI)	*P* value Fisher exact test
Nantes	Se, BIF: 2000 Se, BOOF: 1660 Sp, BIF: 2000 Sp, BOOF: 2000	85.8 (84.3-87.3)	74.9 (72.8-77)	<.001	99.3 (98.9-99.7)	99.8 (99.6-100)	.03
Rennes	Se, BIF: 2000 Se, BOOF: 500 Sp, BIF: 2000 Sp, BOOF: 2000	97.8 (97.1-98.4)	87.6 (84.7-90.5)	<.001	98.2 (97.6-98.7)	99.8 (99.5-100)	<.001
Lille	Se, BIF: 2000 Se, BOOF: 1700 Sp, BIF: 2000 Sp, BOOF: 2000	98.3 (97.7-98.9)	86.8 (85.2-88.4)	<.001	99.4 (99-99.7)	99.4 (90-99.7)	>.99

^a^Se: sensitivity.

^b^Max total distance used: 2

^c^BIF: patient born in France.

^d^BOOF: patient born out of France.

^e^Sp: specificity.

### Application of the Nantes BDW

Among the 1,974,786 (97%) patients recorded as living in the Nantes BDW, 205,698 (10.4%) were matched to the FNMD. Table 7 presents the sex repartition among these patients, and Table 8 presents the age at death by sex. Among all patients linked to the FNMD, 117,563 (57%) were men, and they died 8 years earlier than women did (age 74 years vs 82 years, respectively).

**Table 7:** Sex of patients recorded as living in the Nantes biomedical data warehouse (BDW) and linked to the French National Mortality Database (FNMD).

Sex	Patients in Nantes BDW^a^ (N=205,698), n (%)
Women	88,090 (42.82)
Men	117,563 (57.15)
Unknown	45 (0.022)

^a^BDW: biomedical data warehouse.

**Table 8:** Age at death of patients recorded as living in the Nantes biomedical data warehouse (BDW) and linked to the French National Mortality Database (FNMD).

Variable	Women (N=88,090)	Men (N=117,563)	Unknown (N=45)
Death age (years), median (IQR)	82 (20)	74 (21)	69 (21)

### Large-scale Testing

On our laptop, the execution time to match 200 patients from BDW with the FNMD was 3 minutes, and it was 78 hours to match 2,000,000 patients from BDW with the FNMD. The execution time per patient decreased with the total number of patients. Details are available in Multimedia Appendix 7. The use of blocking techniques reduced the number of required comparisons by at least 40,000 times.

### Open-Access R Code

The R package for the DLD algorithm, called Inseehop, is open access on GitLab [15] and will be maintained and updated by the authors.

## Discussion

### Background

We developed a large-scale DLD-based record-linkage algorithm to match patients from BDWs in France with the FNMD. We then compared the algorithm’s performances with those of a direct-matching algorithm for 3 samples from the Lille, Nantes, and Rennes BDWs.

### Performances That Increased Sensitivity/Recall and Reduced Differential Biases

Overall, sensitivity/recall was approximately 11% higher with the DLD-based algorithm than with the direct algorithm. This highlights the importance of advanced data cleaning and knowledge of a naming system through DLD use.

Moreover, sensitivity was approximately 12% higher for the Lille and Rennes evaluation samples than for the Nantes sample, possibly owing to differences in the BDW data quality or less efficient death report management by regional city halls. Hence, when possible, each center interested in reusing our algorithm should compute its own FNMD-matching performance evaluation.

Sensitivity was approximately 3% lower for women than for men in the Nantes and Rennes samples. This may have been because women are more likely to change their surname after marriage, whereas most men do not; thus, women’s birth surnames are not always registered. The 2020 Réferentiel d’Identitovigilance National Identity Monitoring Guidelines in France [16] recommendusing patients’ birth surnames, even for married women. These differences should consequently disappear in the future.

Sensitivity was higher for people born in France than for those born outside France. This result was expected because other countries’ administrations do not send death reports to INSEE when their citizens die on their territory. Another explanation is that the same surname, first name, or middle name of a non-French patient can have multiple translations in French.

These sensitivity differences (per sex, birth country, or hospital) must be considered when performing an analysis to avoid differential biases between groups.

Finally, increasing the maximal total DLD in the DLD-based algorithm reduced performance gaps between men and women and patients born in and outside of France, which helped limit the differential biases between groups. Specificity was ≥98% for both sexes, birth country, and hospital, which greatly reduced the risk of differential biases between groups.

Blocking the birth date and then concatenating the first 4 characters of the first name and the first 4 characters of the surname reduced the time needed to match 2 million patients from ~366 years to 78 hours. However, records from the local database cannot be compared with those from the FNMD if both these blocking criteria differ; they can only be compared if only 1 differs or both are equal.

### Death Prevalence Was Greatly Underestimated in the BDWs

Applying the DLD-based algorithm to the Nantes BDW revealed that >200,000 patients registered as alive were actually linked to the FNMD, which was approximately 3 times more than the 60,000 patients initially registered as deceased. More men died, and at younger ages, which is consistent with the actual demographic data discussed earlier in this paper.

### Large-scale Matching on a Daily Routine Basis With Minimal Local Computing Capabilities

The program implemented in R software to work on parallel cores was able to run with 2 million patients from the Nantes BDW and 11 million deceased people from the FNMD on 15 GB of RAM and 3 cores in a reasonable duration. Execution time could be improved with higher performance platforms; our laptop was not ideal due to its low computing capabilities and overheating problems during the 3 cores calculations. Because data stayed on hospital computers and no external service was involved, confidentiality was optimal. Moreover, only popular R packages were necessary to run it, which is useful for users who lack administrator rights on their machines.

### Quality of the Gold Standard

As described earlier in this paper, our gold standard was reliable both for sensitivity and specificity evaluation because (1) it was completely independent from the FNMD, (2) deceased status in the hospital databases was reliable, and (3) alive status at a certain time was searched between 2 distinct encounters. For some patients in our sample, names, surnames, birth date, or sex may have been incorrect, as in every database. Nevertheless, this was not a problem because our algorithm could manage these kinds of errors.

### Additional Use Cases

Although our algorithm was originally conceived for linking a large-scale BDW with the large-scale FNMD, it can be used for other purposes, such as matching a large hospital database with an insurance database.

### Limitations

Initially, the expected sample size to evaluate performance at each center was 8000. However, in some cases, there were too few patients with a registered birth country to obtain 1000 patients per strata per center, particularly for WBOF. Nevertheless, sample sizes were sufficient to yield small confidence intervals and significant *P* values.

Another limitation was our methodology, which likely overestimated the sensitivity. Deaths of patients that occurred both inside and outside the hospital and were then communicated to the hospital were not representative of all deceased people. The only way to improve the gold standard would be to conduct an individual investigation of vital status for every patient, which is not possible without significant resources on a large scale.

### Conclusions

While matching operations using names are sensitive computational operations, the Inseehop package we released is easy to run on premises, facilitating compliance with local cybersecurity frameworks. The use of advanced deterministic matching algorithm such as the DLD-based algorithm is an insightful example of combining open-source external data to improve the usage value of BDWs.

## Appendix

Multimedia Appendix 1 Characteristics of the data cleaning and algorithms.

Multimedia Appendix 2 Damerau-Levenshtein distances.

Multimedia Appendix 3 Choice of the most pertinent FNMD records for one local database record in three consecutives steps.

Multimedia Appendix 4 Performance per sex, birth country and maximal total DLD for the DLD-based algorithm in Nantes.

Multimedia Appendix 5 Repartition of the different DLD pair types for a maximal total distance of 5 for the Nantes sample sensitivity estimation.

Multimedia Appendix 6 Repartition of the different DLD pair types for a maximal total distance of 5 for the Nantes sample specificity estimation.

Multimedia Appendix 7 Total execution time and execution time per 10,000 patients.

## Abbreviations

BDW: biomedical data warehouse
DLD: Damerau-Levenshtein distance
INSEE: Institut National de la Statistique et des Études Économiques
FNMD: French National Mortality Database
MBIF: men born in France
MBOF: men born outside France
SNDS: Système National des Données de Santé
WBIF: women born in France
WBOF: women born outside France
