# Supplementary material for: Linking Biomedical Data Warehouse Records With the National Mortality Database in France: Large-scale Matching Algorithm
Source: JMIR Med Inform. 2022 Nov 1;10(11):e36711. doi: 10.2196/36711 (PMC9667378; doi:10.2196/36711)
Supplement: Multimedia Appendix 1 [file medinform_v10i11e36711_app1.doc]

Multimedia Appendix 1: Characteristics of the data cleaning and algorithms

|  | Data cleaning | | Algorithm | | | | | | |
| --- | --- | --- | --- | --- | --- | --- | --- | --- | --- |
|  | Removal of accents and transformation of letters in lowercase^1^ | Removal of numbers and special characters^1^ Advanced data cleaning of birth date^1^ Advanced data cleaning of birth city name^1^ Creation of new variables^2^ | Matching principal of the same variable between the two databases^3^ | Handling of surname^3^ | Management of composed first names^3^ | Blocking on name and birth date^3^ | Choice of the more pertinent pairs^4^ | Package used | Fine tuning of sensitivity/specifity |
| Minimal Data Cleaning + Direct Matching Algorithm | Yes | No | Exact matching | Birth surname if present or current surname if not. | No | No | No | dplyr 1.0.6 | Not possible |
| Advanced Data Cleaning + DLD^a^ based Matching Algorithm | Yes | Yes | DLD^a^ based matching | Birth surname and/or current surname. | Yes | Yes | Yes (optional use of the city name) | inseehop 0.9 | Possible |

^a^DLD: Damerau Lenvensthein Distance

1, 2, 3, 4: first, second, third and fourth step of the DLD algorithm
