# Supplementary material for: Linking Biomedical Data Warehouse Records With the National Mortality Database in France: Large-scale Matching Algorithm
Source: JMIR Med Inform. 2022 Nov 1;10(11):e36711. doi: 10.2196/36711 (PMC9667378; doi:10.2196/36711)
Supplement: Multimedia Appendix 2 [file medinform_v10i11e36711_app2.docx]

Multimedia Appendix 2 : Damerau-Levenshtein distances (DLD)

The DLD between two strings is the minimal number of operations necessary to transform a string (string1) into another (string2). The following operations are allowed:

- insertion of a character
- deletion of a character
- substitution of one character for another in one location
- transposition of two adjacent characters

Many combinations of these four operations may be able to convert string1 to string2, but the length of the shortest sequence is the DLD between the two strings.

For example:

- The DLD between “cabel” and “cabele” is one, because one insertion, the “e”, is enough to transform the first string into the second string.
- The DLD between “demontmiral” and “montmiral” is two, because two deletions, the “d” and the first ”e”, are enough to transform the first string into the second string.
- The DLD between “dupont” and “dupond” is one, because one substitution, the “t” for the “d”, is enough to transform the first string into the second string.
- The DLD between “durand” and “druand” is one, because one transposition, the “r” and the “u”, is enough to transform the first string into the second string.
- The DLD between “weber” and “vuiber” is three, because two substitutions, the “w” in “v” and the “e” in “u”, and one insertion “i” is enough to transform the first string into the second string
