# Supplementary material for: Linking Biomedical Data Warehouse Records With the National Mortality Database in France: Large-scale Matching Algorithm
Source: JMIR Med Inform. 2022 Nov 1;10(11):e36711. doi: 10.2196/36711 (PMC9667378; doi:10.2196/36711)
Supplement: Multimedia Appendix 3 [file medinform_v10i11e36711_app3.docx]

Multimedia Appendix 3: Choice of the most pertinent FNMD records for one local database record in three consecutives steps.

After the blocking processes, a three-step algorithm was applied to every record pair found:

1. If only one record from the FNMD is linked to the local database record, keep this pair and stop the selection. Otherwise, go to step 2.
2. If at least one DLD (first name, family name, birthdate, sex) equals zero, keep all FNMD pairs with at least one DLD equal to 0 and stop the selection. Otherwise, go to step 3a if the birth city option is used or to step 3b if not.

3a) Keep all pairs for which the birth city DLD was the lowest.

3b) Keep all pairs for which the total DLD is the lowest.

Steps 3a and 3b are only possible when the max total DLD chosen equals the sum of the first name, surname, birthdate and sex DLDs. That was not the case for the settings used in our performance evaluation.
