# Supplementary material for: Linking Biomedical Data Warehouse Records With the National Mortality Database in France: Large-scale Matching Algorithm
Source: JMIR Med Inform. 2022 Nov 1;10(11):e36711. doi: 10.2196/36711 (PMC9667378; doi:10.2196/36711)
Supplement: Multimedia Appendix 4 [file medinform_v10i11e36711_app4.docx]

Multimedia Appendix 4 : Performance per sex, birth country and maximal total DLD for the DLD-based algorithm in Nantes.

| max_total_dld | | sex | | Country of birth | | Type | | Value-95% Confidence Interval | |
| --- | --- | --- | --- | --- | --- | --- | --- | --- | --- |
| 0 | | m | | france | | sensitivity | | 86.3 [84.2–88.4] | |
| 0 | | m | | outside_france | | sensitivity | | 70.4 [67.6–73.2] | |
| 0 | | f | | france | | sensitivity | | 81.7 [79.3–84.1] | |
| 0 | | f | | outside_france | | sensitivity | | 70.2 [66.7–73.6] | |
| 0 | | m | | france | | specificity | | 99.8 [99.5–100] | |
| 0 | | m | | outside_france | | specificity | | 99.9 [99.7–100] | |
| 0 | | f | | france | | specificity | | 99.9 [99.7–100] | |
| 0 | | f | | outside_france | | specificity | | 99.9 [99.7–100] | |
| 1 | | m | | france | | sensitivity | | 87.5 [85.5–89.5] | |
| 1 | | m | | outside_france | | sensitivity | | 74.2 [71.5–76.9] | |
| 1 | | f | | france | | sensitivity | | 83.6 [81.3–85.9] | |
| 1 | | f | | outside_france | | sensitivity | | 73.9 [70.6–77.3] | |
| 1 | | m | | france | | specificity | | 99.5 [99.1–99.9] | |
| 1 | | m | | outside_france | | specificity | | 99.9 [99.7–100] | |
| 1 | | f | | france | | specificity | | 99.5 [99.1–99.9] | |
| 1 | | f | | outside_france | | specificity | | 99.9 [99.7–100] | |
| 2 | | m | | france | | sensitivity | | 87.6 [85.6–89.6] | |
| 2 | | m | | outside_france | | sensitivity | | 75 [72.3–77.7] | |
| 2 | | f | | france | | sensitivity | | 84 [81.7–86.3] | |
| 2 | | f | | outside_france | | sensitivity | | 74.8 [71.5–78.2] | |
| 2 | | m | | france | | specificity | | 99.2 [98.6–99.8] | |
| 2 | | m | | outside_france | | specificity | | 99.8 [99.5–100.1] | |
| 2 | | f | | france | | specificity | | 99.4 [98.9–99.9] | |
| 2 | | f | | outside_france | | specificity | | 99.8 [99.5–100] | |
| 3 | | m | | france | | sensitivity | | 87.6 [85.6–89.6] | |
| 3 | | m | | outside_france | | sensitivity | | 75 [72.3–77.7] | |
| 3 | | f | | france | | sensitivity | | 84.1 [81.8–86.4] | |
| 3 | | f | | outside_france | | sensitivity | | 74.8 [71.5–78.2] | |
| 3 | | m | | france | | specificity | | 99.2 [98.6 – 99.8] | |
| 3 | | m | | outside_france | | specificity | | 99.8 [99.5–100] | |
| 3 | | f | | france | | specificity | | 99.3 [98.8–99.8] | |
| 3 | | f | | outside_france | | specificity | | 99.8 [99.5–100] | |
| 4 | | m | | france | | sensitivity | | 87.6 [85.6–89.6] | |
| 4 | | m | | outside_france | | sensitivity | | 75.1 [72.4–77.8] | |
| 4 | | f | | france | | sensitivity | | 84.2 [81.9–86.5] | |
| 4 | | f | | outside_france | | sensitivity | | 74.8 [71.5–78.2] | |
| 4 | | m | | france | | specificity | | 99.2 [98.6–99.8] | |
| 4 | | m | | outside_france | | specificity | | 99.7 [99.4–100] | |
| 4 | | f | | france | | specificity | | 99.2 [98.6–99.8] | |
| 4 | | f | | outside_france | | specificity | | 99.8 [99.5–100] | |
| 5 | | m | | france | | sensitivity | | 87.6 [85.6–89.6] | |
| 5 | | m | | outside_france | | sensitivity | | 75.1 [72.4–77.8] | |
| 5 | | f | | france | | sensitivity | | 84.2 [81.9–86.5] | |
| 5 | | f | | outside_france | | sensitivity | | 74.8 [71.5–78.2] | |
| 5 | | m | | france | | specificity | | 99.2 [98.6–99.8] | |
| 5 | | m | | outside_france | | specificity | | 99.7 [99.4–100] | |
| 5 | | f | | france | | specificity | | 99 [98.4–99.6] | |
| 5 | | f | | outside_france | | specificity | | 99.8 [99.5–100] | |
|  | |  | |  | |  | |  | |
|  |  | |  | |  | |  | |  |
