# Supplementary material for: Linking Biomedical Data Warehouse Records With the National Mortality Database in France: Large-scale Matching Algorithm
Source: JMIR Med Inform. 2022 Nov 1;10(11):e36711. doi: 10.2196/36711 (PMC9667378; doi:10.2196/36711)
Supplement: Multimedia Appendix 5 [file medinform_v10i11e36711_app5.docx]

Multimedia Appendix 5 : Repartition of the different DLD pair types for a maximal total distance of 5 for the Nantes sample sensitivity estimation. N deceased = 3600; n linked at least once = 2963*

| DLD first name | DLD family name | DLD birthdate | DLD sex | Total DLD | N* | Ncum | % | %cum |
| --- | --- | --- | --- | --- | --- | --- | --- | --- |
| 0 | 0 | 0 | 0 | 0 | 2857 | 2857 | 90.9 | 90.9 |
| 0 | 0 | 1 | 0 | 1 | 96 | 2953 | 3.05 | 93.95 |
| 1 | 0 | 0 | 0 | 1 | 49 | 3002 | 1.56 | 95.51 |
| 0 | 1 | 0 | 0 | 1 | 32 | 3034 | 1.02 | 96.53 |
| 0 | 1 | 1 | 0 | 2 | 24 | 3058 | 0.76 | 97.3 |
| 2 | 0 | 1 | 1 | 4 | 18 | 3076 | 0.57 | 97.87 |
| 2 | 0 | 0 | 0 | 2 | 17 | 3093 | 0.54 | 98.41 |
| 1 | 0 | 1 | 1 | 3 | 15 | 3108 | 0.48 | 98.89 |
| 2 | 0 | 1 | 0 | 3 | 8 | 3116 | 0.25 | 99.14 |
| 1 | 0 | 1 | 0 | 2 | 7 | 3123 | 0.22 | 99.36 |
| 1 | 1 | 0 | 0 | 2 | 5 | 3128 | 0.16 | 99.52 |
| 1 | 1 | 1 | 1 | 4 | 5 | 3133 | 0.16 | 99.68 |
| 2 | 1 | 1 | 1 | 5 | 4 | 3137 | 0.13 | 99.81 |
| 1 | 1 | 1 | 0 | 3 | 2 | 3139 | 0.06 | 99.87 |
| 2 | 1 | 0 | 1 | 4 | 2 | 3141 | 0.06 | 99.94 |
| 1 | 0 | 0 | 1 | 2 | 1 | 3142 | 0.03 | 99.97 |
| 2 | 1 | 1 | 0 | 4 | 1 | 3143 | 0.03 | 100 |

* The same person in the BDW can be linked to many records in the FNMD.
