# Supplementary material for: Linking Biomedical Data Warehouse Records With the National Mortality Database in France: Large-scale Matching Algorithm
Source: JMIR Med Inform. 2022 Nov 1;10(11):e36711. doi: 10.2196/36711 (PMC9667378; doi:10.2196/36711)
Supplement: Multimedia Appendix 6 [file medinform_v10i11e36711_app6.docx]

Multimedia Appendix 6: Repartition of the different DLD pair types for a maximal total distance of 5 for the Nantes sample specificity estimation. N alive = 4000; N linked at least once = 23*

| DLD first name | DLD surname | DLD birthdate | DLD sex | Sum all DLD | N | Ncum | % | %cum |
| --- | --- | --- | --- | --- | --- | --- | --- | --- |
| 0 | 0 | 1 | 0 | 1 | 8 | 8 | 32 | 32 |
| 0 | 1 | 1 | 0 | 2 | 6 | 14 | 24 | 56 |
| 0 | 0 | 0 | 0 | 0 | 5 | 19 | 20 | 76 |
| 2 | 1 | 1 | 1 | 5 | 2 | 21 | 8 | 84 |
| 1 | 0 | 1 | 1 | 3 | 1 | 22 | 4 | 88 |
| 2 | 0 | 1 | 0 | 3 | 1 | 23 | 4 | 92 |
| 2 | 0 | 1 | 1 | 4 | 1 | 24 | 4 | 96 |
| 2 | 1 | 1 | 0 | 4 | 1 | 25 | 4 | 100 |

* The same person in the BDW can be linked to many records in the FNMD.
