# Supplementary material for: Linking Biomedical Data Warehouse Records With the National Mortality Database in France: Large-scale Matching Algorithm
Source: JMIR Med Inform. 2022 Nov 1;10(11):e36711. doi: 10.2196/36711 (PMC9667378; doi:10.2196/36711)
Supplement: Multimedia Appendix 7 [file medinform_v10i11e36711_app7.docx]

Multimedia Appendix 7: Total execution time and execution time per 10,000 patients per number of local patients to match to 11 million persons in the French National Mortality Database on three cores and 15 GB of RAM.

| Number of patients to match in the Nantes BDW | Number of cores used | Total execution time (hours) | execution time per 10,000 patients (hours) |
| --- | --- | --- | --- |
| 200 | 1 | 0.05 | 2.63 |
| 200 | 2 | 0.14 | 7.00 |
| 200 | 3 | 0.18 | 8.77 |
| 2000 | 1 | 0.31 | 1.55 |
| 2000 | 2 | 0.28 | 1.42 |
| 2000 | 3 | 0.30 | 1.50 |
| 20000 | 1 | 2.54 | 1.27 |
| 20000 | 2 | 1.56 | 0.78 |
| 20000 | 3 | 1.46 | 0.73 |
| 200000 | 3 | 9.43 | 0.47 |
| 2000000 | 3 | 78.0 | 0.39 |
